# Acute diarrhea in adults consulting a general practitioner in France during winter: incidence, clinical characteristics, management and risk factors

**DOI:** 10.1186/s12879-014-0574-4

**Published:** 2014-10-30

**Authors:** Christophe Arena, Jean Pierre Amoros, Véronique Vaillant, Katia Ambert-Balay, Roxane Chikhi-Brachet, Nathalie Jourdan-Da Silva, Laurent Varesi, Jean Arrighi, Cécile Souty, Thierry Blanchon, Alessandra Falchi, Thomas Hanslik

**Affiliations:** Laboratory of Virology, EA7310, University of Corsica, Corte, France; INSERM, UMR_S 1136, Institut Pierre Louis d’Epidémiologie et de Santé Publique, Paris, F-75013 France; Regional Observatory of Health of Corsica, Ajaccio, France; Department of Infectious Diseases, Institut de Veille Sanitaire (InVS) (French Institute for Public Health Surveillance), Saint-Maurice, France; National Reference Center for Enteric Viruses, Laboratory of Virology, CHU of Dijon, Dijon, France; French National Agency for Research on AIDS and Viral Hepatitis, Paris, France; Sorbonne Universités, UPMC Univ Paris 06, UMRS 1136, Institut Pierre Louis d’Epidémiologie et de Santé Publique, Paris, F-75013 France; Internal Medicine Department, Assistance Publique-Hôpitaux de Paris, Ambroise Paré Hospital, Boulogne Billancourt, F-92100 France; UFR des Sciences de la Santé Simone-Veil, Université de Versailles - Saint-Quentin-en-Yvelines, Versailles, F-78280 France

**Keywords:** Diarrhea, General practice, Adults, Surveillance, Gastroenteritis, Norovirus, Rotavirus

## Abstract

**Background:**

Data describing the epidemiology and management of viral acute diarrhea (AD) in adults are scant. The objective of this study was to identify the incidence, clinical characteristics, management and risk factors of winter viral AD in adults.

**Methods:**

The incidence of AD in adults during two consecutive winters (from December 2010 to April 2011 and from December 2011 to April 2012) was estimated from the French *Sentinelles* network. During these two winters, a subset of *Sentinelles* general practitioners (GPs) identified and included adult patients who presented with AD and who filled out a questionnaire and returned a stool specimen for virological examination. All stool specimens were tested for astrovirus, group A rotavirus, human enteric adenovirus, and norovirus of genogroup I and genogroup II. Age- and sex-matched controls were included to permit a case–control analysis with the aim of identifying risk factors for viral AD.

**Results:**

During the studied winters, the average incidence of AD in adults was estimated to be 3,158 per 100,000 French adults (95% CI [2,321 – 3,997]). The most reported clinical signs were abdominal pain (91.1%), watery diarrhea (88.5%), and nausea (83.3%). GPs prescribed a treatment in 95% of the patients with AD, and 80% of the working patients with AD could not go to work. Stool examinations were positive for at least one enteric virus in 65% (95% CI [57 – 73]) of patients with AD with a predominance of noroviruses (49%). Having been in contact with a person who has suffered from AD in the last 7 days, whether within or outside the household, and having a job (or being a student) were risk factors significantly associated with acquiring viral AD.

**Conclusions:**

During the winter, AD of viral origin is a frequent disease in adults, and noroviruses are most often the cause. No preventable risk factor was identified other than contact with a person with AD. Thus, at the present time, reinforcement of education related to hand hygiene remains the only way to reduce the burden of disease.

**Electronic supplementary material:**

The online version of this article (doi:10.1186/s12879-014-0574-4) contains supplementary material, which is available to authorized users.

## Background

In industrialized countries, acute diarrhea (AD) is a major cause of morbidity and medical expenses, particularly in vulnerable populations, such as elderly patients who are more often hospitalized, stay in the hospital longer and die more often than younger individuals when AD occurs [1]. Infectious AD can be caused by various microbiological pathogens such as bacteria, parasites or viruses. AD occurs year-round but exhibits a pronounced winter peak, related to an increase in AD of a viral origin, mainly due to noroviruses and group A rotavirus infections [2]-[4].

Few studies have described the epidemiology and management of viral AD in adults during the winter. During the winter of 1998–1999 in France, human caliciviruses were shown to be the most frequently encountered viruses in 16- to 65-year-old patients consulting a general practitioner (GP) for AD, while group A rotavirus predominated in patients 65 years of age and older [2]. During the 1995–1996 winter, risk factors shown to be associated with AD in France included contact with a person with AD, living with a child ≤2 years of age, and recent treatment with oral penicillin or cephalosporin [5]. However, in this study, microbiological investigations were not required, and the results were presented for all age groups and not specifically for adults. In the Netherlands, hand hygiene and contact with a sick person were identified as risk factors for viral gastroenteritis related to caliciviruses and group A rotavirus infections, but approximately 90% of the included patients were <10 years of age [6]. The management of viral AD in general practice was studied for rotavirus infections in children [7], but to our knowledge, such data are not available for viral AD occurring in adults.

Thus, data describing the epidemiology and management of viral AD in adults seen in general practice are scant. The objective of this study was to identify the clinical characteristics, management and risk factors associated with the occurrence of viral AD in French adults consulting a GP.

## Methods

### Study design

In France, continuous surveillance of AD is conducted by the French *Sentinelles* GPs network (www.sentiweb.fr) [8],[9]. *Sentinelles* GPs’ characteristics, such as regional distribution, proportion in rural practice, type of practice and types of main clinical skills, are comparable to those of all French GPs [10].

The study was conducted over two consecutive winters from the 49^th^ week of 2010 (2010w49) to 2011w17 and then from 2011w49 to 2012w17.

The *Sentinelles* GPs reported (via the Internet) information regarding all adult individuals (≥18 years old) presenting with AD, which was defined as “at least 3 daily watery (or nearly so) stools, less than 14 days”. The age and sex of the patients were documented.

A sample of *Sentinelles* GPs participated in a complementary survey with the aim of investigating clinical characteristics, virology, and management of AD occurring in adults. They were asked to recruit one AD case per week. To ensure that the selection of patients remained random, the GP had to include the first patient seen in consultation and who met the inclusion criteria in that particular week. Patients with inflammatory bowel disease and patients with an obvious non-viral etiology of diarrhea (traveler’s diarrhea, recent use of antibiotics, colchicine, non-steroidal anti-inflammatory drugs or laxatives, or recent administration of chemotherapy or radiotherapy) were excluded.

*Sentinelles* GPs were also asked to include one age- and sex-matched patient per AD case for a nested case–control study. The study’s aim was to identify the risk factors associated with the occurrence of viral AD. This matched individual presented just after the AD case for a non-gastrointestinal disease and did not report any gastrointestinal symptoms during the month preceding the consultation.

The GPs completed and sent a case report form for all patients included in the complementary survey by postal mail. The case report included collected data on gender, age and potential risk factors. The studied risk factors were factors related to lifestyle (professional status, educational level, presence in the household of children ≤2 years of age, contact with pets or farm animals, hand hygiene, suffering from a chronic disease), and exposure during the last 7 days (contact with persons with AD in and/or outside the household; having eaten an unusual meal; consumption of tap water, oysters, mussels or shellfish; having used public transport; and/or having gone to a swimming pool). Data on reported symptoms, medications, days of missed work, additional medical examinations, or required hospitalizations were also collected for each AD case.

Patients included in the complementary survey were asked to collect and send stool specimens by postal mail in triple packaging (according to the United Nations class 6.2 specifications). They were also asked to return a follow-up questionnaire the week after enrollment to indicate the duration of symptoms (AD patients) and to ascertain whether an AD had occurred or not (non-AD patients).

### Virological analysis

All stool specimens were tested for four enteric viral pathogens (astrovirus, group A rotavirus, human enteric adenovirus, and norovirus of genogroup I - NoVGI - and genogroup II - NoVGII) using the Seeplex® Diarrhea-V ACE assay (Seegene) according to the manufacturer’s instructions. A recent study showed that the Seeplex® Diarrhea-V assay is a sensitive, specific, convenient and reliable method to simultaneously detect several viral pathogens found directly in stool specimens from patients with gastroenteritis [11].

### Statistical analysis

The AD cases reported via the Internet by the *Sentinelles* GPs allowed the estimation of winter incidence rates for mainland France by age group (18 – 39 years, 40 – 59 years, 60 – 79 years and ≥80 years). The winter incidence rate was calculated as follows: the average number of cases notified by *Sentinelles* GPs (adjusted for participation and geographic distribution) was multiplied by the total number of private GPs practicing in France and then divided by the French population [12],[13]. Confidence intervals were estimated by assuming that the distribution of the number of reported cases followed a Poisson distribution.

The data collected during the complementary survey were entered twice to ensure consistency. Data analysis was performed using STATA (version 11.2, StataCorp LP, Texas, USA). Quantitative variables were described by using medians [interquartile range IQ] and means – standard deviations and were compared by the Wilcoxon test. Qualitative variables were described by using proportions and compared using a chi-square or Fisher’s exact test if the chi-square test were not applicable; the results were presented as odds ratio with 95% confidence intervals (OR [95% CI]).

For the nested case–control study, a *case* was a patient with AD in which at least one enteric virus was identified; a *control* was a matched patient without AD in which no enteric virus was identified. Univariate analyses were conducted using the McNemar test. A conditional logistic regression model was used to study the independent effects of risk factors that were associated in the univariate analyses (p-value of <0.20). Variables for the model were chosen through automatic backwards selection using a significance level of 0.05. Assuming a control-to-case ratio of 1:1, an exposure rate of 15% among controls, a two-tailed level of significance of 5% and a power level of 80%, 87 cases were needed to detect a minimal odds ratio (OR) of 3.

### Ethics statement

Oral consent was obtained from the patients at the time of inclusion for their participation in the study and for the publication of the clinical and virological data.

The Hospital Ethics Committee (CHU Saint-Antoine, Paris, France) approved the study.

## Results

### Incidence rates in general practice

**Figure 1:**
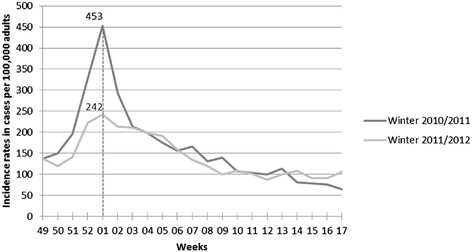
**Weekly incidence rates of acute diarrhea in adults (≥18 years old) consulting a GP in France (estimated using the French** ***Sentinelles*** **GPs network).**

During the two winters studied, 370 GPs participated in the electronic surveillance, and 10,415 AD cases were reported. Figure 1 shows the weekly incidence rates, and Table 1 presents the winter incidences and incidence rates by age groups. The median age of adult patients seen by the *Sentinelles* GPs over the two consecutive winters was 37 years (IQ = [27 – 52]) and 36 years (IQ = [27 – 51]), respectively; the proportion of men was 46.2% and 45.4% over the two winters, respectively.

**Table 1:** **Incidence rates of acute diarrhea in France by age group per 100,000 cases estimated by the French** ***Sentinelles*** **GPs network during two consecutive winters**

	Winter 2010/2011		Winter 2011/2012	
	2010w49 – 2011w17		2011w49 – 2012w17	
	Incidence [IC 95%]	Incidence rate per 100,000 [IC 95%]	Incidence [IC 95%]	Incidence rate per 100,000 [IC 95%]
18 years of age and older	1,691,959 [1,287,372 – 2,096,954]	3,471 [2,641 – 4,302]	1,472,351 [1,060,343 – 1,885,771]	3,002 [2,162 – 3,845]
*18 – 39 years*	953,943 [784,323 – 1,123,563]	5,388 [4,430 – 6,346]	859,608 [678,755 – 1,040,461]	4,920 [3,885 – 5,956]
*40 – 59 years*	470,312 [351,703 – 588,921]	2,771 [2,072 – 3,470]	390,424 [273,691 – 5 07,157]	2,290[1,606 – 2,975]
*60 – 79 years*	194,142 [122,101 – 266,248]	1,761 [1,107 – 2,415]	162,982 [90,142 – 235,822]	1,420 [785 – 2,055]
*≥ 80 years*	73,562 [29,245 – 118,222]	2,200 [875 – 3,536]	59,337 [17,755 – 102,331]	1,668 [499 – 2,876]

### Clinical characteristics, management and virology

Among the 100 *Sentinelles* GPs who agreed to participate in the complementary survey, 65 enrolled 192 adult patients who were seen for AD. Their median age was 36 years (IQ = [28 – 52]), and 111 (57.8%) were men. The reported clinical signs are presented in Table 2.

**Table 2:** **Reported clinical signs in adult patients consulting a GP for acute diarrhea (complementary survey)**

	Patients with acute diarrhea (N = 192) (%)
Average time before consultation ± sd (days)	1.6 ± 1.8
Average duration of diarrhea ± sd (days)	2.0 ± 1.8
Average number of stools in the last 24 h ± sd	5.7 ± 2.8
Average max. number of stools per day ± sd	6.0 ± 2.9
Mucous diarrhea	29 (15.1%)
Bloody diarrhea	2 (1.0%)
Watery diarrhea	170 (88.5%)
Abdominal pain	175 (91.1%)
Nausea	160 (83.3%)
Vomiting	119 (62.0%)
*Average duration* ± *sd (days)*	*1.0 ± 1.1*
Fever	83 (43.2%)
*Average body temperature* ± *sd (°C)*	*38.4 ± 0.5*
*Average duration* ± *sd (days)*	*1.4 ± 1.3*
Dehydration	8 (4.2%)
Other symptoms	8 (4.2%)

Overall, 183 (95.3%) patients received a drugs prescription, which were mostly intestinal antisecretory drugs (N = 98, 53.6%), antiemetics (N = 96, 52.4%), antispasmodics (N = 72, 39.3%), intestinal adsorbents (N = 65, 35.5%), analgesics/antipyretics (N = 54, 29.5%) and regulators of intestinal motility (N = 54, 29.5%). Among the 146 patients who reported having a job, 116 (79.5%) benefited from stopping work for a median duration of 3 days (IQ = [2 – 3]), regardless of gender.

Stool samples from 145 patients with AD (75.5%) were returned. The median age of those patients was 37.5 years (IQ = [30 – 54]), and 80 were men (55.2%). Stools tested positive for at least one of the four enteric viruses investigated in 94 cases (65%). The detailed results from the virological investigation are presented in Table 3. Among the patients with AD, the reported clinical signs did not differ between adults with a virus in the stool sample and those with no virus found in the stool exam, neither in frequency nor in severity (Table 4). Thus, the management of patients with AD who tested positive for a virus was not different from the management of patients who tested negative (data not shown). None of the cases required hospitalization.

**Table 3:** **Results from the virological investigation of adult patients consulting a general practitioner for acute diarrhea in France from week 2010w49 to week 2011w17 and from week 2011w49 to week 2012w17 (complementary survey)**

Viruses detected	Patients with acute diarrhea (N = 145) (%)
Norovirus GII	59 (40.7)
Norovirus GI	17 (11.7)
Astrovirus	5 (3.5)
Rotavirus	2 (1.4)
Adenovirus 40/41	0 (0.0)
Coinfections	11 (8.0)
*Norovirus GI + GII*	*5 (3.5)*
*Norovirus GI + Rotavirus A*	*1 (0.7)*
*Norovirus GI + Astrovirus*	*1 (0.7)*
*Norovirus GII + Astrovirus*	*4 (2.8)*
At least one virus detected	94 (64.8)
No virus detected	51 (35.2)

**Table 4:** **Reported clinical signs in adult patients consulting a GP for acute diarrhea in virus-positive and virus-negative stool samples (complementary survey)**

	Patients with acute diarrhea		
	At least one virus detected (N = 94) (%)	No virus detected (N = 51) (%)	p-value*
Average age ± sd (years)**	40.4 ± 15.1	44.3 ± 17.8	0.16
Men**	52 (55.9%)	28 (57.1%)	0.89
Average time before consultation ± sd (days)	1.6 ± 1.9	1.7 ± 1.9	0.92
Average duration of diarrhea ± sd (days)	1.6 ± 1.5	2.1 ± 1.8	0.23
Average number of stools in the last 24 h ± sd	5.4 ± 2.7	6.3 ± 3.3	0.09
Average max. number of stools per day ± sd	5.7 ± 2.5	6.5 ± 3.3	0.13
Mucous diarrhea	10 (11.0%)	8 (17.4%)	0.30
Bloody diarrhea	1 (1.1%)	1 (2.2%)	0.63
Watery diarrhea	83 (91.2%)	39 (84.8%)	0.26
Abdominal pain	85 (93.4%)	42 (91.3%)	0.66
Nausea	77 (82.8%)	36 (78.3%)	0.52
Vomiting	61 (66.3%)	24 (52.2%)	0.11
*Average duration* ± *sd (days)*	*0.8 ± 0.9*	*1.3 ± 1.4*	*0.12*
Fever	42 (46.2%)	15 (33.3%)	0.16
*Average body temperature* ± *sd (°C)*	*38.3 ± 0.4*	*38.5 ± 0.7*	*0.42*
*Average duration* ± *sd (days)*	*1.2 ± 1.2*	*1.7 ± 1.7*	*0.30*
Dehydration	3 (3.3%)	1 (2.2%)	0.71
Other symptoms	5 (5.5%)	1 (2.2%)	0.39

*Logistic regression: adjustment for age and sex.

**Not adjusted for age and sex.

### Risk factors for viral AD

The GPs enrolled 101 matched individuals for the nested case–control study. Among them, 95 patients mailed back a stool specimen. Of the stools examined, 4 tested positive (4.2%) for one enteric virus (NoVGII) and were excluded from the case–control study. Thus, 91 pairs (51 male and 40 female) were included in the analysis. The median age was 36 years (IQ = [28 – 50]) for the cases and 37 years (IQ = [29 – 53]) for the controls. Viral acute diarrheas were independently associated with having been in contact with a person who has suffered from an AD in the last 7 days, either within or outside the household, and having a job or student (Table 5). The contact of cases with sick people outside the household had taken place either at work (59%) or other place (41%). The median duration between the contact with a sick person and the onset of the symptoms was 2 days (IQ = [1 – 4]).

**Table 5:** **Factors associated with viral acute diarrhea (cases) in 91 pairs of adult patients consulting a GP**

	Cases (N = 91) (%)	Controls (N = 91) (%)	OR uni [95% CI] (p-value)*	OR multi [95% CI] (p-value)*
Professional status (employed or student/non employed or retired)	80 (87.9%)	67 (73.6%)	4.25 [1.43 – 12.63] (0.01)	4.10 [1.27 – 13.21] (0.02)
Educational level (high school and above/middle school)	80 (87.9%)	69 (75.8%)	2.83 [1.12 – 7.19] (0.03)	2.37 [0.86 – 6.57] (0.10)
Children ≤2 years in household (yes/no)	20 (22.0%)	9 (9.9%)	2.57 [1.07 – 6.16] (0.03)	1.87 [0.69 – 5.09] (0.22)
Being in contact with pets or farm animals (yes/no)	45 (49.5%)	48 (52.8%)	0.80 [0.48 – 1.58] (0.65)	n.i.
Washing hands before cooking (never-sometimes/often-always)	10 (11.6%)	13 (16.1%)	0.58 [0.23 – 1.48] (0.26)	n.i.
Washing hands after using the toilet (never-sometimes/often-always)	7 (7.7%)	8 (8.8%)	0.86 [0.29 – 2.55] (0.78)	n.i.
Washing hands after attending public places (never-sometimes/often-always)	45 (52.3%)	35 (40.7%)	1.50 [0.76 – 2.95] (0.24)	n.i.
Suffering from a chronic disease (yes/no)	28 (30.8%)	29 (31.9%)	0.94 [0.48 – 1.86] (0.86)	n.i.
Contact with persons with AD in the household (yes/no)	31 (34.1%)	10 (11.0%)	5.20 [2.00 – 13.50] (0.01)	4.18 [1.54 – 11.33] (<0.01)
Contact with persons with AD outside household (yes/no)	22 (24.2%)	9 (9.9%)	3.60 [1.34 – 9.70] (0.01)	3.31 [1.03 – 10.63] (0.04)
Having eaten an unusual meal (yes/no)	33 (36.3%)	28 (30.8%)	1.39 [0.68 – 2.83] (0.37)	n.i.
Having consumed oysters, mussels, or shellfish (yes/no)	27 (29.7%)	29 (31.9%)	0.90 [0.48 – 1.70] (0.75)	n.i.
Having consumed tap water (yes/no)	69 (75.8%)	73 (80.2)	0.76 [0.37 – 1.58] (0.47)	n.i.
Having used public transportation (yes/no)	20 (22.0%)	14 (15.4%)	2.00 [0.75 – 5.33] (0.17)	2.57 [0.71 – 9.39] (0.15)
Going to a public swimming pool (yes/no)	4 (4.4%)	5 (5.5%)	0.80 [0.22 – 2.98] (0.74)	n.i.

*Conditional logistic regression: matched for age and sex.

OR: odds-ratio; Uni: univariate; Multi: multivariate; CI: confidence interval; n.i: not included in the multivariate model.

## Discussion

This study presents the first analysis of the global burden of AD in adults who consulted a GP in France. Winter incidences, clinical characteristics, virological investigation, management and risk factors for viral AD were investigated.

### Incidence rates in general practice

During the two studied winters, 3,471 and 3,002 cases per 100,000 French adults consulted a GP for an AD in winters 2010/2011 and 2011/2012, respectively. The data on AD incidences vary from country to country because of differences in case definition, surveillance systems, and/or the period of study. In France, a telephone survey estimated the incidence rate of acute gastroenteritis at 0.33 cases/person-year [14]. In the Netherlands, a population-based study conducted in 1998/1999 estimated that the gastroenteritis incidence was 283 per 1,000 person-years [15]. In both studies, the incidence rate peaked in children and then decreased in adults.

### Clinical characteristics, management and virology

In this study, more than 80.0% of patients reported abdominal pain, watery diarrhea, and/or nausea, while vomiting and fever were reported by 62.0% and 43% of patients, respectively. These results are in agreement with other French studies [2],[14].

Adults are less likely to consult a GP for gastroenteritis compared with children, as it remains a self-limiting disease [14]. Patients with more severe symptoms are more prone to consulting a GP, which is illustrated by the fact that 80% of working adult cases had to stop working. Although no cases required hospitalization, the economic burden of AD related to outpatient visits could be significant, because the average annual incidence of AD in adults is 1 million cases (www.sentiweb.fr). In addition to the cost of outpatient visits, medical treatment and missed work days increase the heavy burden of viral AD cost in adults. Indeed, 95% of the patients in this study received a drug prescription. The management of AD is most likely amenable to a more appropriate drug prescription in France. For example, antiemetics are prescribed in a majority of cases, whereas their efficacy in this indication has never been validated, and their side effects may be serious [16].

The feces samples were not screened to rule out bacterial and parasitic infections. However, we included patients in whom there was a very high suspicion of viral diarrhea (and a very low risk of bacterial or parasitic infection), as inclusions were done during winter and cases with an obvious non-viral etiology of diarrhea were excluded. During the winter, viral AD is predominant, but the reason is not clear. Hypotheses for these findings include that the clustering of people indoors during the winter months facilitates person-to-person transmission and the enhanced persistence of noroviruses at low temperatures [17]. Noroviruses have been described as the leading cause of winter AD [18], and the GII genogroup strains have been previously shown to predominate during winter, although the reason for this remains unclear [19]. In this study, the proportion of adult patients with AD who were positive for at least one enteric virus (65%) was higher than in previous studies in general practice performed in France or Europe (15-39%) [2]-[4]. However, unlike these studies, the aim of our study was to generate a sample of patients who were positive for a virus; thus, patients with obvious non-viral diarrhea were excluded. In the patients included here, the clinical characteristics of AD, and thus its management, were not different for adults with or without an identified virus in the stool. It is possible that a study with more statistical power would have identified some clinical differences, such as more frequent occurrence of vomiting [20].

### Risk factors for viral AD

Being previously in contact with an individual presenting with AD was identified as a risk factor for developing AD. Norovirus and rotavirus are among the most communicable pathogens responsible for AD. Experimentally, an inoculum as low as 500 (and even less) viable organisms is sufficient to establish an infection, and the virus is environmentally stable [20]. Thus, enteric viruses have a high potential for person-to-person spread. The increased risk in people who have had a contact with a sick person in the household is consistent with this already well-known mode of transmission [21]. In 1995 and 1996 in France, Lettrillard et al. [5] showed that the risk of developing AD was 5 times higher in patients who had been in contact with a person suffering from AD in their household. However, the study included patients whose AD etiology was unknown (no stool sample). Studies in Germany [4] and the Netherlands [6] have confirmed this observation and estimated adjusted ORs ranging from 1.9 to 12.9 based on viral detection; however, the results of these studies were not stratified by age. In this study, patients who reported having a job and students were significantly more likely to suffer from viral AD than those who were unemployed or retired. Among the studies that have tried to identify the risk factors for acquiring an AD, none have investigated professional status. The result obtained in this study seems quite relevant and suggests that this population has increased contact with sick people, which is the main risk factor for infection. The acquisition of a viral AD may be associated with other factors that were not identified in this study. For example, De Wit et al. showed that norovirus AD risk was increased in people with poorer hand hygiene (OR = 1.3 [1.0 – 1.7]) [6], which was not observed here. It has also been shown that living with children ≤2 years of age increases the risk of developing AD in the winter, regardless of the children’s health status (AD or not) [5]. The association between developing AD and living with children ≤2 years that was identified in our univariate analysis did not persist after adjusting for other variables. No association between viral AD and tap water use, seafood consumption or an unusual meal was found.

## Conclusions

During the winter, AD of viral origin is a frequent disease in adults with a significant burden in the population. Noroviruses are mainly responsible for the disease. Other than contact with a person suffering from AD, no other preventable risk factor was identified. Thus, at the present time, education related to hand hygiene remains the only way to reduce the burden of disease.

## Authors’ contributions

CA, JPA, VV, KAB, RCB, NJDS, LV, JA, TB, AF and TH co-conceived the study. CA and TH collected the epidemiological and microbiological data. AF, LV and KAB designed the microbiology experiments. AF performed the microbiology experiments and analyzed and interpreted the data. CA and CS analyzed and interpreted the statistical data. CA, JPA, VV, KAB, RCB, NJDS, LV, JA, CS, TB, AF and TH contributed to writing the paper and approved the final manuscript.

## Authors’ original submitted files for images

Below are the links to the authors’ original submitted files for images. Authors’ original file for figure 1

## Abbreviations

AD: Acute Diarrhea
GP: General Practitioner
NoVGI: Norovirus of Genogroup I
NoVGII: Norovirus of Genogroup II
(RT)-PCR: (Reverse Transcription)-Polymerase Chain Reaction
IQ: InterQuartile range
OR: Odds Ratio
95% CI: 95% Confidence Interval
