# Supplementary figures and images for: Acute diarrhea in adults consulting a general practitioner in France during winter: incidence, clinical characteristics, management and risk factors
Source: BMC Infect Dis. 2014 Oct 30;14:574. doi: 10.1186/s12879-014-0574-4 (PMC4220050; doi:10.1186/s12879-014-0574-4)

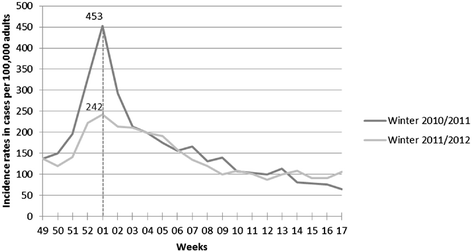

Supplement: Supplementary file 1 — Authors’ original file for figure 1 [file 12879_2014_574_MOESM1_ESM.gif]
